# Baicalin Enhances Chemosensitivity to Doxorubicin in Breast Cancer Cells via Upregulation of Oxidative Stress-Mediated Mitochondria-Dependent Apoptosis

**DOI:** 10.3390/antiox10101506

**Published:** 2021-09-23

**Authors:** Mei-Yi Lin, Wan-Ting Cheng, Hui-Ching Cheng, Wan-Ching Chou, Hsiu-I Chen, Hsiu-Chung Ou, Kun-Ling Tsai

**Affiliations:** 1 Department of Chinese Medicine, Ditmanson Medical Foundation Chia-yi Christian Hospital, Chiayi 600, Taiwan; 04129@cych.org.tw; 2 Department of Food Nutrition and Health Biotechnology, College of Medical and Health Science, Asia University, Taichung 413, Taiwan; 3 Department of Physical Therapy, College of Medicine, National Cheng Kung University, Tainan 701, Taiwan; s98094015@gs.ncku.edu.tw (W.-T.C.); 10710014@gs.ncku.edu.tw (H.-C.C.); T66051040@pt.ncku.edu.tw (W.-C.C.); 4 Institute of Clinical Medicine, College of Medicine, National Cheng Kung University, Tainan 701, Taiwan; 5 Department of Physical Therapy, College of Medical and Health Science, Asia University, Taichung 413, Taiwan; chenpt@sunrise.hk.edu.tw; 6 Department of Physical Therapy, Hungkuang University, Taichung 433, Taiwan; 7 Institute of Allied Health Sciences, College of Medicine, National Cheng Kung University, Tainan 701, Taiwan

**Keywords:** doxorubicin, baicalin, chemosensitivity, ROS, apoptosis

## Abstract

Doxorubicin (Dox) is an effective anthracycline anticancer drug. However, recent studies have revealed that Dox resistance is a highly critical issue, and a significant reason for treatment failure. Baicalin is a flavonoid component in the roots of *Scutellaria baicalensis* Georgi; however, whether baicalin can increase chemosensitivity in breast cancers is still unclear. In this study, we found that cellular apoptosis occurs when excessive intracellular ROS is generated, triggered by the dual intervention of baicalin and doxorubicin, which increases intracellular calcium [Ca^2+^]_i_ concentrations. Increased [Ca^2+^]_i_ concentrations decrease the mitochondrial membrane potential (△Ψ_m_), thereby causing cellular apoptosis. Pretreatment with NAC (ROS inhibitor) or BATBA (Ca^2+^ chelator) reduces baicalin-induced chemosensitivity. The findings of this study demonstrate that the effect of baicalin on Dox treatment could enhance cytotoxicity toward breast cancer cells via the ROS/[Ca^2+^]_i_-mediated intrinsic apoptosis pathway—thus potentially lessening the required dosage of doxorubicin, and further exploring associated mechanisms in combined treatments for breast cancer clinical interventions in the future.

## 1. Introduction

Doxorubicin (Dox), one of the most effective anticancer drugs in the anthracycline class, is constantly utilized in the clinical treatment of breast cancer; nevertheless, it contributes to the irreversible side-effect of cardiotoxicity—characterized by the loss of myofibrils, dilated sarcoplasmic reticulum, swelling of mitochondria, cytoplasmic vacuolization, and increased number of lysosomes, which is a species-independent final morphologic impairment [1]. Dox is taken as a single-agent treatment, with an average response rate of around 50% and a reported maximal response rate of 80% [2]. However, recent studies have revealed that Dox resistance is one of the most critical issues in current treatments, and a significant reason for treatment failure [3,4].

Many compounds extracted from natural products present chemopreventive properties and show a potent sensitization function in variant cancer cells [5]. Baicalin (C_21_H_18_O_11_; 5,6,7-trihydroxyflavone 7-*O*-beta-d-glucuronide) is one of the flavonoid components present in the roots of *Scutellaria baicalensis* Georgi, with a concentration ranging from 8.1% to 15.6% [6,7]. Numerous studies have claimed that baicalin possesses pharmacological properties comprising anti-inflammatory, anticancer, antithrombotic, cardioprotective, and antioxidant effects by depressing the activity of NF-κB and stifling the expression of several inflammatory cytokines and chemokines [8,9,10]. Baicalein, an aglycone of baicalin, has been explored for its many effects, including effects on apoptosis through the ERK/p38 MAPK pathway, suppression of adhesion, migration, and invasion in breast cancer cells, and attenuation of oxidant stress in cardiomyocytes; however, there may be other similar mechanisms or yet undiscovered mechanisms underlying baicalin’s activities [9,11,12].

The extent of baicalin’s effects when combined with chemotherapeutic agents is still unknown. We assumed that treatment using a combination of baicalin and Dox would enhance the efficacy of the drug by increasing apoptosis and cellular function in breast cancer cells. Combining baicalin with Dox could represent a new therapeutic strategy, with the potential to promote the anticancer capacity of Dox.

## 2. Materials and Methods

### 2.1. Reagents

**Figure 1:**
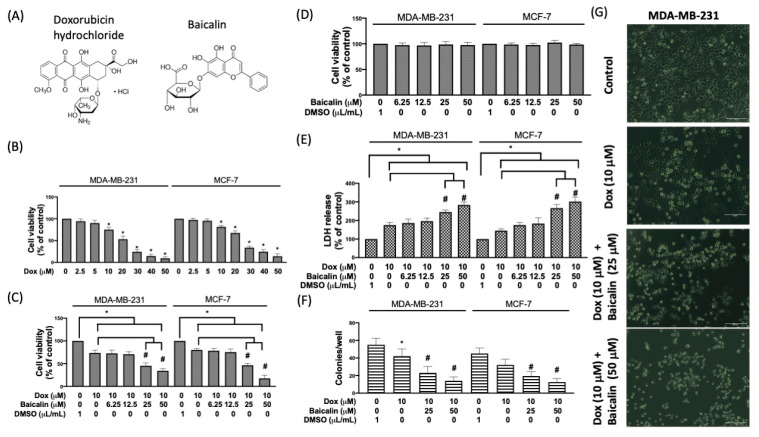
Baicalin promotes doxorubicin (Dox)-reduced cell viability and cell survival under Dox treatment in MDA-MB-231 and MCF-7 breast cancer cells. (**A**) Structures of dox and baicalin. (**B**) MDA-MB-231 breast cancer cells were incubated with Dox (2.5–50 μM) for 24 h; viability was detected using the MTT assay. (**C**) Cotreatment of 10 µM Dox with various concentrations of baicalin. (**D**) A single infusion of baicalin did not reduce the cell viability. (**E**) Cell cytotoxicity was determined according to the level of released lactate dehydrogenase (LDH) from cells. (**F**) Quantification of single-cell clone proliferation after Dox treatment. (**G**) Cells express blebbing membranes, where a shrunken or rounded and condensed morphology was more common following treatment with 10 µM Dox and was further induced in baicalin-coincubated cells. Data are expressed as the mean ± SEM of three independent analyses; * *p* < 0.05 versus untreated control, ^#^ *p* < 0.05 versus Dox treatment.

Fetal bovine serum, MEM nonessential amino acids, sodium pyruvate, Dulbecco’s modified Eagle’s medium, trypsin-EDTA, and penicillin were obtained from Gibco (Grand Island, New York, NY, USA). The LDH cytotoxicity assay kit, superoxide dismutase activity assay kit, and CaspGLOW™ red active caspase-3 staining kit were obtained from BioVision (Palo Alto, CA, USA). The in situ cell death detection kit, fluorescein, doxorubicin hydrochloride, 5,6,7-rrihydroxyflavone (baicalin), anti-β-actin, 3-(4,5-cimethylthiazol-2-yl)-2,5-diphenyl tetrazolium bromide (MTT), 5,5′,6,6′-tetrachloro-1,1′,3,3′-tetraethyl-imidacarbocyanine iodide (JC-1), and 2′,7′-dichlorofluorescein diacetate (DCF-DA) were purchased from Sigma (St. Louis, MO, USA). Figure 1A reveals the structures of doxorubicin and baicalin. Anti-Bax, anti-Bad, anti-Bcl-2, and anti-cytochrome c were acquired from Cell Signaling Technology (Danvers, MA, USA). Fura-3 AM was obtained from Thermo Fisher Scientific (Waltham, MA, USA).

### 2.2. Cell Culture

Human triple-negative breast cancer cells MDA-MB-23, MCF-7 human breast cancer cells, and H9C2 cardiomyoblast cells were purchased from the American Type Culture Collection (ATCC, Manassas, VA, USA) and were cultured in Dulbecco’s modified Eagle’s medium (DMEM) containing 10% (*v*/*v*) fetal bovine serum (FBS), 100 IU/mL penicillin, 10 mM MEM nonessential amino acids (NEAA), and 100 mM sodium pyruvate. Cells were incubated in a humidified atmosphere with 5% CO₂ at 37 °C. To obtain the Dox-resistant phenotype, MDA-MB-231and MCF-7 cells were exposed to DMEM with 5% FBS with increasing concentrations of doxorubicin (10 nM to 100 nM). After 3 weeks, the P0 Dox-resistant cells were selected.

### 2.3. Investigation of Colony Formation

MDA-MB-231 and MCF-7 cells were seeded at a density of 1 × 10^5^ cells/well in 6 cm dishes for 24 h in an incubator with 5% CO₂. Then, the cells were treated with Dox (10 µM) with or without baicalin coincubation for 24 h. After incubation for 24 h, the cells were trypsinized and seeded in 3.5 cm dishes at a density of 800 cells/well, and then cultured in high-glucose DMEM with 10% FBS for 10 days at 37 °C. Colonies were stained with 1% crystal violet for 15 min at RT, and the number of colonies was counted under a microscope.

### 2.4. Determination of Cell Viability, Cytotoxicity, and Apoptosis

The cell viability of MDA-MB-231 cells seeded in 24-well plates with a concentration of 5 × 10^4^ cells/well was assessed by adding 250 µL of MTT and incubating in a humidified atmosphere with 5% CO₂ at 37 °C for 1 h, followed by adding 250 µL of dimethyl sulfoxide (DMSO) after MTT was removed. Then, 100 µL of supernatant from each sample was transferred to a 96-well plate before measuring absorbance at a wavelength of 540 nm. The cytotoxicity of cells was evaluated using lactate dehydrogenase (LDH) with an LDH cytotoxicity assay kit to measure the absorbance of all samples at a wavelength of 495 nm. Apoptotic cells were determined by the terminal deoxynucleotidyl transferase-mediated dUTP nick end labeling (TUNEL) assay using flow cytometry.

### 2.5. Measurement of ROS Production and Antioxidant Enzyme Activity

The 2′,7′-dichlorofluorescein diacetate (DCF-DA) probe was used to gauge intracellular ROS. Cells were supplemented with DCF-DA/medium at a ratio of 1:1000 and were gently shaken in the dark for 30 min, followed by removing the supernatant after centrifuging at 1300 rpm for 3 min. Then, 500 µL of phosphate-buffered saline (PBS) was added to analyze intracellular ROS via flow cytometry. In addition, cells maintained in 24-well plates were complemented with DCF-DA/medium at 37 °C for 30 min, before examining intensity (excitation/emission wavelength = 504/524 nm) under a fluorescence microscope. Superoxide dismutase (SOD) activity was tested using a SOD activity kit.

### 2.6. Measurement of Calcium Concentration

After treatment with Dox or Dox plus baicalin, Fura-3 AM (μg/mL) was added to cells in the dark for 30 min. Cells were washed with PBS three times and de-attached using trypsin-EDTA. Fluorescence intensity was assayed by flow cytometry.

### 2.7. Immunoblotting

Cells were lysed in RIPA lysis buffer and centrifuged at 13,000 rpm for 20 min. Cellular proteins from the supernatant were separated by electrophoresis on SDS-polyacrylamide gel, followed by transferring proteins onto a polyvinylidene difluoride membrane prior to blocking the blots with 1× PBS and 5% nonfat dry milk for 1 h at room temperature. Primary antibodies were introduced for probing at 4 °C overnight, followed by incubation with HRP-conjugated secondary antibodies (1:5000) for 1 h and detection by chemiluminescent HRP substrate (Millipore Inc., Billerica, MA, USA).

### 2.8. Measurement of Active Caspase-3

Caspase-3 activity is increased associated with the protease cascade during apoptosis. To explore caspase-3 activity induced by the combination of doxorubicin and baicalein, cells detected via flow cytometry were processed using the CaspGLOW™ red active caspase-3 staining kit according to the manufacturer’s instructions (BioVision Inc., Palo Alto, CA, USA).

### 2.9. Measurement of Mitochondrial Membrane Potential (△Ψ_m_)

The sensitive and relatively mitochondrion-specific lipophilic cationic probe fluorochrome 5,5′,6,6′-tetrachloro-1,1′,3,3′-tetraethyl-imidacarbocyanine iodide (JC-1) was added to explore the mitochondrial membrane potential in breast cancer cells. Depolarized mitochondria prevent JC-1 mitochondrial entry and incite its disaggregation, leading to diffuse green fluorescence (excitation wavelength = 520 nm); conversely, in the presence of a hyperpolarized membrane potential, JC-1 forms red-fluorescent (emission wavelength = 596 nm) “J-aggregates”, which exhibit broad excitation and very narrow emission spectra. Cells were evaluated via flow cytometry and fluorescence microscopy.

### 2.10. Statistical Analyses

Results are expressed as the mean ± standard error of the mean (SEM). Differences between the groups were analyzed using Student’s *t*-test. A *p*-value of <0.05 was considered statistically significant. All data were analyzed using SPSS software version 22.0 (Ins., Chicago, IL, USA).

## 3. Results

### 3.1. Baicalin Increased Dox-Impaired Cell Viability and Reduced Cell Survival under Dox Treatment in MDA-MB-231 and MCF-7 Breast Cancer Cells

Human triple-negative breast cancer cells (MDA-MB-231) were treated with various concentrations of doxorubicin (50, 40, 30, 20, 10, 5, or 2.5 µM) for 24 h. The 10 µM Dox concentration reduced cell viability to around 70% (Figure 1B). Thus, the final concentration of 10 µM Dox was used to test the potential for increasing chemosensitivity in MDA-MB-231 cells. Figure 1C reveals that 50 µM and 25 µM baicalin significantly reduced cell viability compared to Dox-only cells. However, 50 µM and 25 µM baicalin treatment without coincubation with Dox did not reduce the viability of MDA-MB-231 breast cancer cells (Figure 1D). The LDH assay revealed that 50 µM and 25 µM baicalin promoted Dox-induced cell death and cytotoxicity (Figure 1E). In addition to affecting cell viability, we also found that Dox reduced the formation of colonies compared with the control group. Compared to the Dox group, the formation of colonies was also significantly reduced by Dox plus 50 µM and 25 µM baicalin (Figure 1F). Moreover, we also investigated the morphological changes in breast cancer cells stimulated with 10 µM Dox. Figure 1G shows that 25 µM and 50 µM baicalin treatments plus Dox stimulation exhibited more shrunken or condensed breast cancer cells.

### 3.2. Baicalin Enhanced Dox-Induced Oxidative Stress in MDA-MB-231 and MCF-7 Breast Cancer Cells

**Figure 2:**
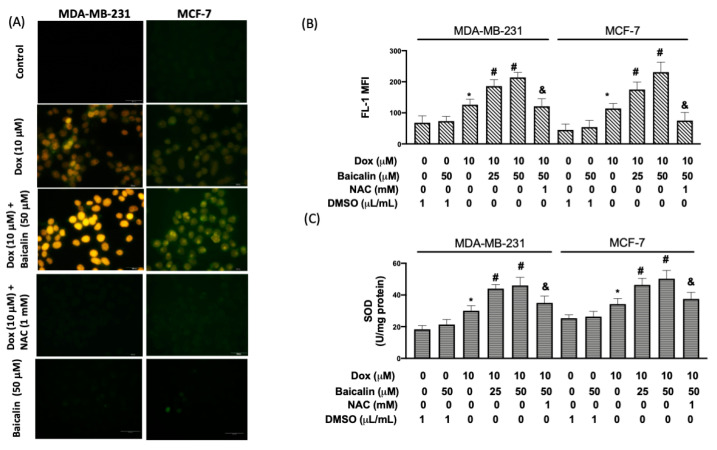
Baicalin enhances Dox-induced oxidative stress in MDA-MB-231 and MCF-7 breast cancer cells. Cells were treated with 10 μM Dox or 10 μM Dox plus 50 or 25 µM baicalin for 24 h. DCF–DA was used to study ROS levels. Images are shown under a fluorescence microscope (**A**). ROS levels (mean fluorescence intensity; MFI) were investigated by flow cytometry (**B**). SOD activity was mitigated in cells treated with Dox and baicalin; in some cells, NAC pretreatment inhibited oxidative stress (**C**). Data are expressed as the mean ± SEM of three independent analyses; * *p* < 0.05 versus untreated control, ^#^ *p* < 0.05 versus Dox treatment, ^&^ *p* < 0.05 versus Dox+Baicalin (50 μM) treatment.

Various studies have reported the Dox-induced generation of reactive oxygen species (ROS) in the clinical treatment of breast cancer as a central mediator of direct and indirect cardiac side effects [13]. To define whether intervention with Dox could trigger ROS emergence when combined with baicalin, intracellular ROS was examined using 2′,7′-dichlorofluorescein diacetate (DCF–DA). The results of fluorescence microscopy (Figure 2A) and flow cytometry (Figure 2B) revealed that the combined treatment of Dox and baicalin possessed a stronger effect in terms of ROS levels than 10 µM Dox-only cells, regardless of the dosage of baicalin (25 µM or 50 µM). The overlaid image shows that 25 µM baicalin or 50 µM baicalin did not cause ROS production. Moreover, many have argued that antioxidants, such as superoxide dismutase (SOD), are scavengers of free radicals and are beneficial guardians for several oxidative stress-associated pathologies involving inflammatory reactions, cellular transformation, cellular apoptosis, etc. [14,15]. We found that Dox enhanced the activity of SOD, while Dox plus baicalin (25 µM or 50 µM) further upregulated the activity of SOD compared to Dox-only cells. Pretreatment with NAC (an ROS inhibitor) mitigated activity levels in cells treated with Dox plus 50 µM baicalin—indicating that baicalin enhances Dox-induced oxidative stress by increasing ROS production (Figure 2C).

### 3.3. Baicalin Increased Intracellular Calcium Concentration and Impairment of Mitochondrial Membrane Potential under Dox Treatment

**Figure 3:**
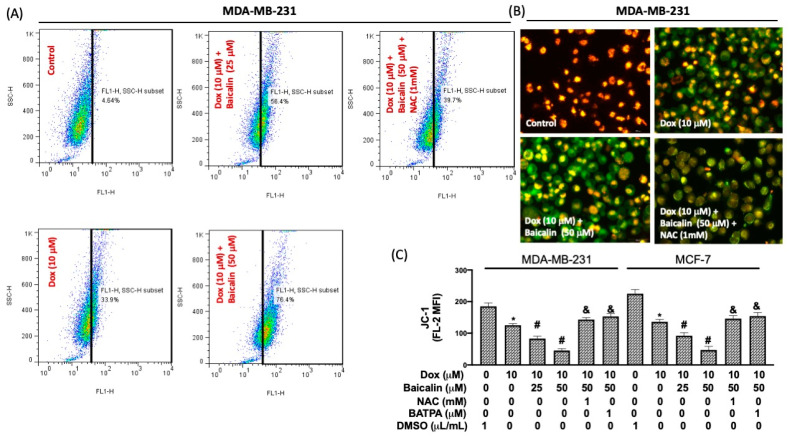
Baicalin increases intracellular calcium concentration and impairs mitochondrial membrane potentials. Cells were treated with 10 μM Dox or 10 μM Dox plus 50 or 25 µM baicalin for 24 h. Fura-3 AM was used to reveal the intracellular calcium concentration by flow cytometry. Combined treatments (baicalin plus Dox) showed a massive increase in the number of positive cells compared to Dox alone. In some cells, NAC pretreatment inhibited oxidative stress (**A**). The mitochondrial membrane potential was investigated using fluorescent microscopy (**B**) and flow cytometry (**C**) with JC-1. Red fluorescence denotes the potential-dependent aggregation of JC-1 within polarized mitochondria in healthy cells, and green fluorescence denotes depolarized mitochondria in unhealthy cells or apoptotic cells. Data are expressed as the mean ± SEM of three independent analyses; * *p* < 0.05 versus untreated control, ^#^ *p* < 0.05 versus Dox treatment, ^&^ *p* < 0.05 versus Dox+Baicalin (50 µM) treatment.

Intracellular calcium [Ca^2+^]_i_ triggers key cellular functions such as metabolic modulation and mitotic division, thereby regulating cell death [16,17,18]. Figure 3A reveals that [Ca^2+^]_i_ levels were higher in Dox plus baicalin cells compared to Dox-only cells. Pretreatment with NAC reduced this phenomenon, suggesting that baicalin elevates [Ca^2+^]_i_ under Dox treatment in breast cancer cells through increased oxidative stress. Moreover, mitochondrial membrane potentials arise from proton pumping across a membrane by the electron transport chain, exhibiting ionic permeability critical to the maintenance of mitochondrial function [19]. The JC-1 probe—a fluorescent dye able to prompt the alteration of mitochondrial membrane potentials during apoptosis [20]—possesses red fluorescence, defining the potential-dependent aggregation of JC-1 within polarized mitochondria in healthy cells, and green fluorescence, identifying depolarized mitochondria in unhealthy cells as a function of the monomeric form of JC-1 in the cytosol [21]. As mentioned above, red fluorescence (FL-2) was clearly lower in Dox plus baicalin cells compared to Dox-only cells (Figure 3B). Pretreatment with NAC or BATBA (Ca^2+^ chelator) reduced the mean fluorescence intensity (MFI) of FL-1 (Figure 3C), suggesting that baicalin promotes the impairment of mitochondrial membrane potentials under Dox treatment through modulation of the ROS/[Ca^2+^]_i_ mechanism.

### 3.4. Baicalin Promoted Apoptosis in Breast Cancer Cells with Dox Treatment and Increased Chemoselectivity in Dox-Resistant Breast Cancer Cells, but Protected Cardiomyoblast Cells from Dox-Induced Apoptosis

**Figure 4:**
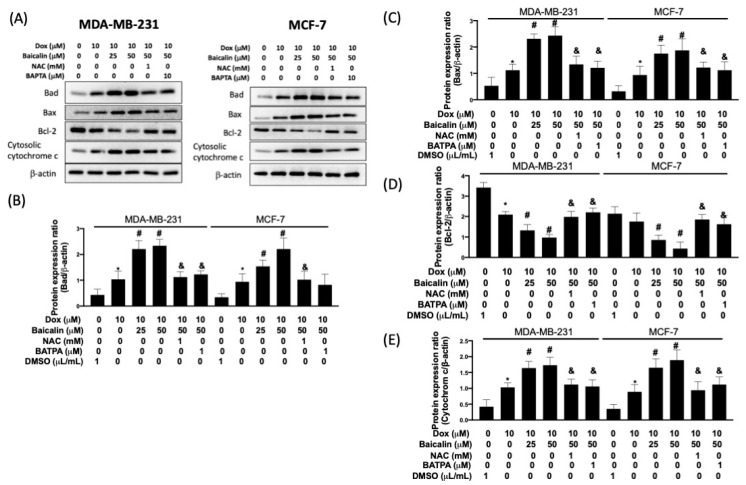
Baicalin promotes Dox-induced apoptosis by mediating the mitochondrial-dependent pathway. Cells were treated with 10 μM Dox or 10 μM Dox plus 50 or 25 µM baicalin for 24 h. In some cells, NAC pretreatment inhibited oxidative stress, whereas BAPTA pretreatment reduced the concentration of intracellular calcium. The expression levels of Bad, Bax, Bcl-2, and cytosolic cytochrome c were investigated using a Western blot assay (**A**). Protein expression levels were quantified and are presented as a bar chart (**B**–**E**). Data are expressed as the mean ± SEM of three independent analyses; * *p* < 0.05 versus untreated control, ^#^ *p* < 0.05 versus Dox treatment, ^&^ *p* < 0.05 versus Dox+Baicalin (50 µM) treatment.

The Bcl-2 family plays a pivotal role in the intrinsic pathway of apoptosis. In Figure 4, the expression of proapoptotic factors, Bad and Bax, was higher in Dox plus baicalin cells compared to Dox-only cells. However, expression levels of Bcl-2 were lower in Dox plus baicalin cells compared to Dox-only cells. In addition, the released cytochrome c sequentially binds to Apaf-1, contributing to the formation of the apoptosome, recruiting cleaved caspase-9 [22]. We found that cytochrome c expression levels were enhanced in Dox plus baicalin cells compared to Dox-only cells. As expected, pretreatment with NAC or BATBA reduced baicalin-promoted apoptosis under Dox treatment, suggesting that baicalin enhances apoptosis under Dox treatment through upregulation of ROS/[Ca^2+^]_i_.

**Figure 5:**
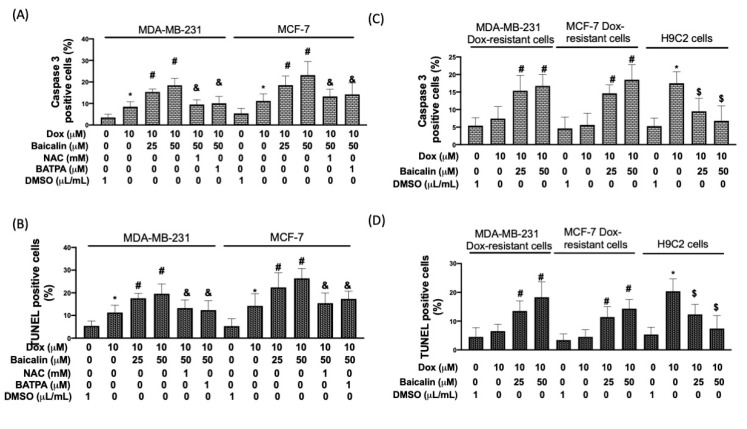
Baicalin increases Dox-induced apoptosis through an ROS/[Ca^+^]_i_ mechanism. Cells were treated with 10 μM Dox or 10 μM Dox plus 50 or 25 µM baicalin for 24 h. In some cells, NAC pretreatment inhibited oxidative stress, whereas BAPTA pretreatment reduced the concentration of intracellular calcium. Apoptotic cells were studied as a function of activated-caspase 3 (**A**) and the TUNEL assay **(B**) in MDA-MB-231 cells, MCF-7 cells and in (**C**,**D**) Dox-resistant cells, MCF-7 Dox-resistant cells, and H9C2 cells. Data are expressed as the mean ± SEM of three independent analyses; * *p* < 0.05 versus untreated control, ^#^ *p* < 0.05 versus Dox treatment, ^$^ *p* < 0.05 (lower) versus Dox treatment, ^&^ *p* < 0.05 versus Dox+Baicalin (50 μM) treatment.

The activation of caspase-3 was detected using flow cytometry. Dox plus baicalin increased the number of caspase-3-positive cells compared to the Dox-only group (Figure 5A). TUNEL enables labeling of the 3′-freehydroxyl ends of the fragmented DNA with fluorescein–dUTP, catalyzed by terminal deoxynucleotidyltransferase [23]; therefore, the TUNEL assay was utilized to further clarify cellular apoptosis in Dox-treated breast cancer cells with or without baicalin. As presented in Figure 5B, Dox plus baicalin increased the number of TUNEL-positive cells compared to the Dox-only group. Pretreatment with NAC or BATBA reduced baicalin-activated caspase-3 and DNA damage. Moreover, to address whether baicalin could increase chemoselectivity in Dox-resistant breast cancer cells, we isolated MDA-MB-231 Dox-resistant cells and MCF-7 Dox-resistant cells by increasing the concentration of Dox in the culture medium. Figure 5B,C reveal that baicalin increased the Dox-induced upregulation of caspase-3 and DNA damage in Dox-resistant breast cancer cells. Moreover, we confirmed that Baicalin did not increase, but instead reduced apoptosis in noncancer cells (cardiomyoblast cells) under Dox stimulation.

## 4. Discussion

Anthracycline agents have been widely utilized in breast cancer treatments; nevertheless, they can lead to adverse effects. To improve the advanced occurrence of side effects during clinical intervention, this study aimed to comodulate breast cancer cells with baicalin and doxorubicin, targeting chemosensitivity toward apoptosis. The outcomes of the study reveal that baicalin could effectively enhance anticancer ability via cellular apoptosis through an ROS/ER stress/mitochondrial intrinsic pathway in stubborn breast cancer cells. Numerous lines of evidence have indicated that DNA-damaging agents or chemotherapeutic agents, such as anthracycline or cisplatin, may induce cell apoptosis in sensitive tumor cells [24]; notwithstanding, there have been some studies claiming resistance to apoptosis as the major mode of drug resistance to antitumor treatments [25]. In this study, we chose the cooperative intervention of baicalin with Dox in an attempt to overcome chemosensitivity via the induction of apoptosis. We found that this approach reduced cell viability, increased ROS and [Ca^2+^]_i_, and enhanced cellular apoptosis in Dox-treated breast cancer cells.

Mitochondria are the most consequential source of intracellular ROS, as their leakage from the electron transfer chain is considered the major route of ROS production [26]. Following the overproduction of reactive oxygen species, a disturbance will be triggered in the endoplasmic reticulum (ER), representing the main source of Ca^2+^ storage—thus initiating apoptosis via the distribution of calcium from the ER to the cytosol and mitochondria.

Ca^2+^, as a second messenger mediating cellular homeostasis, can be transferred by ER Ca^2+^ release channels, IP3 receptors (IP3Rs), and ryanodine receptors (RyRs) [27]. The respiratory chain complexes of the mitochondria allow the translocation of H^+^ in the intermembrane space, thereby generating an electrochemical gradient (ΔµH) containing chemical (ΔpH) and electrical (ΔψH) components, supplying a tremendous driving force for Ca^2+^ entry into the organelle. When excessive ROS are generated, Ca^2+^ migrates to the mitochondria and contributes to the depolarization of the mitochondrial membrane potential (△Ψm) via modulation of the Bcl-2 family, before the release of soluble mitochondrial intermembrane proteins (SIMPs) such as cytochrome c [28,29,30]. Accordingly, we can state that the improved effect of baicalin on Dox in terms of the chemosensitivity of breast cancer cells is due to the induction of apoptosis through an ROS/depolarized mitochondrial membrane potential pathway.

Bcl-2 family members can be subdivided into three series on the basis of their pro- or antiapoptotic function [31,32,33]. Bad interacts with distinct sites in Bax, which can elicit a conformational alteration, thereby contributing to its insertion and oligomerization in the outer mitochondrial membrane (OMM) and stimulating pro-survival Bcl-2 proteins [34,35,36,37]. We found that baicalin promotes Bax and Bad expression levels under Dox treatment compared with Dox-only cells.

In conclusion, we discovered that 25 µM and 50 µM baicalin with 10 µM Dox could heighten the chemosensitivity of MDA-MB-231 and MCF-7 breast cancer cells compared to Dox-only treatment. More intracellular ROS were generated after combined treatment with baicalin and Dox, thereby attenuating superoxide dismutase (SOD) activity. Moreover, baicalin-enhanced oxidative stress further increased the concentration of [Ca^2+^]_i_, thereby modulating cellular apoptosis in breast cancer cells. Our results suggest that baicalin is a promising tool in the mitigation of chemoresistance in breast cancer patients treated with Dox-based interventions.

## Data Availability

The data presented in this study are available in this article.
